# Hyaluronan binding assay (HBA) vs. sperm penetration assay (SPA): Can HBA replace the SPA test in male partner screening before in vitro fertilization?

**Published:** 2010-02-10

**Authors:** Jelena Lazarevic, Maria Wikarczuk, Stephen G. Somkuti, Larry I. Barmat, Jay S. Schinfeld, Scott E. Smith

**Affiliations:** 1 Abington IVF & Genetics, Abington Reproductive Medicine, Abington, PA, 19001, USA; 2 Toll Center for Reproductive Sciences, Abington Memorial Hospital, Abington, PA, 19001, USA

**Keywords:** Hyaluronan-binding assay, sperm penetration assay, male partner screening

## Abstract

**Objective:**

To determine if a less expensive, easier, and faster to perform HBA test is clinically equal to the more complicated, technically challenging and expensive SPA test as a reliable indicator of sperm fertilizing capacity.

**Design:**

Prospective study.

**Setting:**

Andrology laboratory within In Vitro Fertilization Program.

**Patient(s):**

Semen samples from 26 infertility couples were analyzed. Both, normal and male factor patients were included.

**Intervention(s):**

Male partner screening with the HBA and the SPA tests.

**Main Outcome Measure(s):**

Relationship between HBA and SPA test results.

**Result(s):**

The data obtained in this study showed no statistically significant relationship between the HBA and SPA results. The mean HBA scores 76.3%, 61.3% and 76.8% were statistically not significantly different as compared to patients with negative (<5), grey zone (5–8) and for positive (>8) sperm capacitation index values.

**Conclusion(s):**

The HBA is not predictive of the results of the SPA. Therefore, HBA test does not reduce the need for and cannot replace the SPA test in male partner screening prior to infertility treatment.

## Introduction

The most important part of the management of male infertility is a correct diagnosis. The semen analysis is widely performed as a major test of male fertility potential, by assessing sperm count, motility and morphology of the spermatozoa. It is clear that these parameters are not sufficient alone to interpret the fertility status of an ejaculate, unless significantly abnormal. Sperm function may not be predicted by semen analysis, as the fertilization process involves a large number of biochemical events not measured by these parameters. Thus, semen analysis is limited in its inability to assess the fertilizing potential of the sample (1–5). Nearly one third of male factor infertility etiologies remain unexplained and are considered idiopathic (6). Additional tests need to be used to indicate the functional activity of spermatozoa (7, 8). The sperm penetration assay (SPA) is one such test that provides additional information for sperm fertilizing ability, using zona free hamster oocytes (9). Unfortunately, the SPA is costly, technically challenging, time consuming and is not readily performed in many infertility clinics. We chose to examine a less costly, technically easier alternative for assessing sperm function that could serve as a useful screening tool to aid in the decision making process to determine which appropriate reproductive techniques should be used.

The hyaluronan binding assay (HBA) evaluates the maturity of sperm in a fresh semen sample (10). The HBA is a simple technique proposed as a component of the standard semen analysis in the diagnosis of suspected male infertility, to predict sperm performance and fertilization potential. However, diagnostic use of the HBA is still investigational. To further the understanding of the HBA we explore the relationship between the SPA and HBA tests. We investigate the possibility of replacing the SPA (complicated, long and expensive) with the HBA test (simple, short and less costly).

## Materials and Methods

### Patients

The study population included 26 randomly selected male patients with a history of infertility and those who came to andrology laboratory for a first evaluation of fertility potential. Both, normal and male factor patients were included. Institutional review board approved this study. Since all tests were performed on discarded human sperm samples which were coded to avoid any link to the donor, no informed consents were obtained.

### Study Design

Conventional semen analysis with Kruger morphology, hyaluronan binding assay and sperm penetration assay were performed for each male patient on the same semen sample. Routine semen analysis parameters and Kruger morphology values, HBA and SPA test results (SCI) for tested 26 patients are noted in Table 1. The results are presented as mean values (+/−) standard deviations and range of the evaluated test values.

### Conventional Semen Analysis

Ejaculates were collected by masturbation after 2–5 days of abstinence. Each specimen after liquefaction was evaluated with routine semen analysis including sperm count and motility according to guidelines from the World Health Organization, 1999 (3). The Kruger classification for sperm morphology was also recorded (11).

### Hyaluronan Binding Assay (HBA)

Semen samples from 26 infertility patients were also tested with HBA (Biocoat, INC, Ft. Washington, PA, USA), following the manufacturer’s instructions. Each HBA slide had two identical chambers with a molecular layer of hyaluronan covalently attached to the chamber surface. These slides are for determination of the hyaluronan-binding fraction of motile sperm in a semen sample. Mature, motile sperm bind with hyaluronan through specific receptors. We applied 10 μl of semen to a chamber and installed a cover slip, incubated for 10 minutes, at room temperature (18–28ºC) to allow for complete binding. Then under a microscope we counted the numbers of bound and unbound motile sperm in the same number of grid squares, in the same sample volume. Bound motile sperm appeared on the slide showing rapidly beating tails, but their heads showed no forward, progressive movement, while unbound motile sperm swam around freely. The HBA score (%) was calculated as # bound motile sperm / # total motile sperm. The value of the HBA is based on the proposition that a low level of sperm binding to hyaluronan is suggestive that there is a low proportion of mature sperm in the sample (10).

### Methodology of Sperm Penetration Assay (SPA)

The SPA is a multi-step, two day laboratory test. On day one of SPA we used basic method of processing sperm, mixing and overnight incubation in the Test Yolk buffer refrigeration medium (Irvine Scientific, Santa Ana, CA, USA) at 4ºC for 18–20 hours, to slowly effect and induce capacitation (12). On day two of SPA, after sperm capacitated during overnight storage, it was warmed to 37ºC i.e. thermal shocked (12), and sperm concentration was adjusted. On day two of SPA, zona free hamster oocytes were prepared by enzymatic digestion, washed and allowed to incubate 3 hours with sperm at 37ºC under 5% CO2, before determining the percentage of eggs penetrated and the number of sperm penetrations per egg.

### Day One of Sperm Penetration Assay (SPA)

Semen samples were prepared for SPA testing by performing a sperm gradient (45/90) Isolate (Irvine Scientific, Santa Ana, CA, USA) and two washes with IVC (In Vitro Care, Frederick, MD, USA) Human Tubal Fluid (HTF) - Hepes with 10% IVC Human Serum Albumin (HSA). The washed sample was mixed with an equal volume of Test Yolk buffer refrigeration medium and was incubated overnight in the refrigerator at 4ºC for 18–20 hours to allow capacitation.

### Day Two of Sperm Penetration Assay

#### Preparation of Sperm

Following the incubation period, supernatant was removed, and the pellet was resuspended in 2.0 ml of warmed IVC HTF Hepes with 10% HSA (thermal shock) and centrifuged at 300g for 10 minutes. The supernatant was discarded. The pellet was resuspended in 0.1 – 1.0 ml of IVC HTF with 10% HSA and placed in the incubator for 30–60 minutes. An SPA control semen specimen was run in parallel.

#### Preparation of Zona – Free Hamster Oocytes

On day two of SPA hamster oocytes were thawed according to Embryotech Laboratories INC., (Wilmington, MA, USA) package instructions. Hamster oocytes zona were removed for 30–60 seconds at room temperature in 1mg/ml Trypsin (Sigma-Aldrich Corp, St. Louis, MO, USA) and rinsed thoroughly in IVC HTF Hepes with 10% HSA. The 15 zona-free hamster oocytes per dish were inseminated with 3 million motile sperm and placed in the incubator for 3–3.5 hours. After the incubation period any loosely bound sperm were removed. Oocytes were placed in the polyvinylpyrrolidone-PVP (IVF online.com, Guilford, CT, USA) drops on the microscope glass slide. A 24X40 glass cover slip was carefully placed over PVP drops, without entrapping air bubbles. Swollen sperm heads with attached tails were scored as penetrations in each oocyte using Nikon Labophot-2 Light Microscope (40X objective). Sperm capacitation index (SCI) was calculated as the total number of sperm penetrations divided by the number of hamster oocytes analyzed. Normal values established in our laboratory were SCI <5 (negative score), <5 SCI <8 (gray zone) and SCI >8 (positive score).

#### Statistical Analysis

Significance was tested by linear regression, Student’s t-test and the Mann-Whitney U (Mann-Whitney-Wilcoxon) test, with the latter two tests performed using two different cut-off points (SCI=5 and SCI=8).

## Results

**Figure 1:**
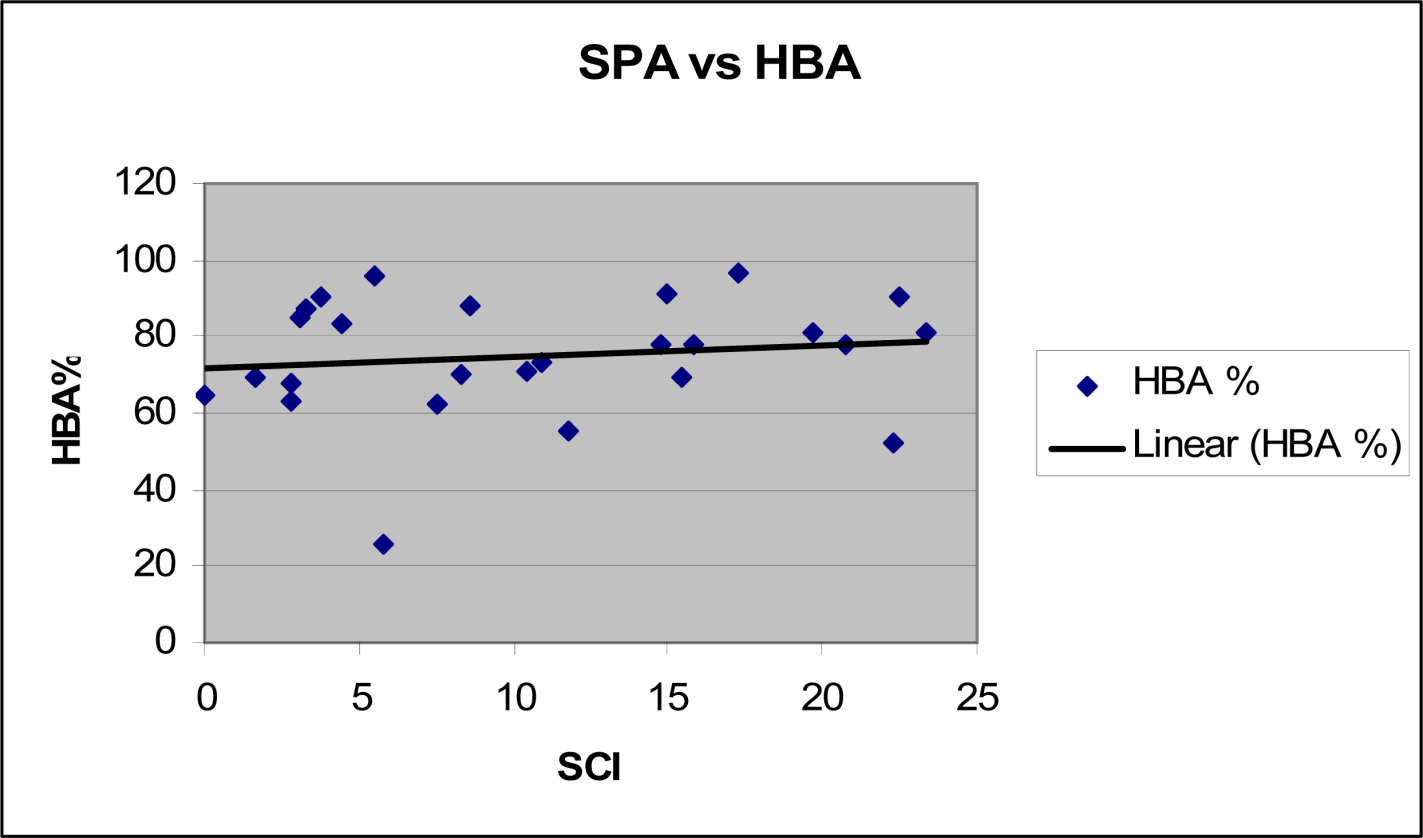
Linear regression analysis of SPA (SCI results) compared with HBA scores (%)

Linear regression analysis (Figure 1) showed no statistically significant relationship between HBA and SPA results (R^2^=0.02, F= 0.51, p=0.48). The mean HBA scores were 76.3%, 61.3% and 76.8% for the patients with negative (n=8), grey zone (n=3) and for positive (n=15) SCI values, respectively. Student’s t-test and the MWW test showed no statistically significant difference between the two pairs of sample means. Taking 5 as the SCI cut-off value, the mean difference between the corresponding HBA sample scores (n1=8, n2= 18) is 2.03 with t=0.30 and p=0.77 (MWW test: z= −0.11 and p=0.91). When the SCI cut-off value is set to 8, the mean difference between the two samples (n1=11, n2=15) is −4.62 with t=−0.74 and p=0.47 (MWW test: z= −0.65 and p=0.52).

## Discussion

Sperm capacitation index – SCI, (the average number of sperm penetrations per hamster egg), was compared with HBA scores, (the number of mature sperm in percent), for 26 patients. Correlation between SPA and HBA tests would be recognized if patients with higher SPA scores had higher HBA scores. However, in our lowest SPA score group (negative SCI<5), the mean HBA score was 76.3%, similar to the HBA score of 76.8% in our highest SPA score group (positive SCI >8). The present study showed that the HBA was not significantly correlated with the SPA results as analyzed by linear regression, Student’s t-test and the Mann-Whitney U (MWW) test.

In order to promote better understanding of our results we briefly explain some of the complicated events involved in the fertilization process. Fertilization occurs when a sperm fuses with an egg to form a zygote (13, 14). Sperm must first be capacitated; that is, their membranes must become fragile so that the hydrolytic enzymes in their acrosomes can be released during acrosome reaction (13, 15). Capacitation requires separation of sperm from seminal plasma, followed by incubation for a defined period in the female tract or an appropriate laboratory medium (16) when spermatozoa undergo a series of biochemical transformations. The ovulated oocyte is encapsulated by the corona radiata and zona pellucida, both of which must be breached before the oocyte itself can be penetrated (13). After breaching the corona, the sperm head binds to the ZP3 glycoprotein of the zona pellucida and causes a rise in Ca^2+^ level within the sperm that triggers the acrosomal reaction (13). Hundreds of sperm acrosomes must rupture to break down the intercellular cement, rich in hyaluronic acid, that holds the granulosa cells together and to digest holes in the zona pelucida (13). Once a path has been cleared and a single sperm makes contact with the oocyte’s membrane receptors, their plasma membranes fuse, allowing sperm nucleus to be pulled into the oocyte’s cytoplasm and chromatin decondensation occurs (13).

The SPA test, in determining the ability of sperm to penetrate zona-free hamster eggs directly, addresses four discrete physiological steps in the process of fertilization: capacitation, acrosome reaction, fusion with and penetration through the oolemma, and decondensation within the cytoplasm of an oocyte (9)

Sperm oocyte fusion in the hamster oocyte penetration test differs from the physiological situation in that the zona pellucida is absent (3). The conventional hamster oocyte test depends upon the occurrence of spontaneous acrosome reactions in populations of spermatozoa incubated for prolonged periods of time in vitro (3).

Preincubation in TEST-yolk buffer at 4ºC makes spermatozoa highly fusogenic, enhancing sperm penetration (17). As hypothesized by Mortimer (18), incubation in TEST-yolk buffer at low temperature promotes intracellular accumulation of Ca^2+^ ions, due to a reduced activity of calcium pump; moreover, the egg yolk phospholipids intercalate into membranes, making the spermatozoa highly labile. However, acrosome reactions are prevented by low temperature. Hence, upon restoring physiological temperature, the elevated Ca^2+^ concentrations trigger synchronized acrosome reactions, enhancing sperm penetration (18).

The SPA preparation method with Test-yolk technique correlates well with fertility (12, 17) and with the outcome of IVF (19).

The SPA has been used for many years for diagnosing and implementing appropriate infertility treatment (19, 20, 21). The SPA allows an important differentiation between patients who can have conventional in vitro insemination-IVF and those who require intracytoplasmic sperm injection-ICSI (22). For many couples with a normal semen analysis and SPA test, maximal fertilization can be achieved with conventional insemination (22). ICSI was introduced as a treatment for severe male infertility. However, some centers consider that ICSI rather than IVF should be offered as the treatment of choice to all cases requiring assisted reproduction (23).

There are numerous studies done on children born after ICSI that have looked at the potential effects of ICSI, with differing conclusions (24, 25, 26, 27). Since the studies are not in agreement, perhaps the safest assumption is that ICSI is not to be used until conventional insemination is highly unlikely to succeed (28).

Even though SPA may be a useful technique for predicting appropriate treatment options (22), widespread adoption of the SPA test has been limited due to the lack of standardization of SPA protocols and experimental conditions among centers (2, 29). Typical capacitation time for sperm incubation before the SPA is 18–20 hours (9). Therefore, inconsistent SPA results may be due to inappropriately short capacitation times (30, 31). Whether due to extreme complexity, cost, or questionable reliability, the reality is that the SPA test is rarely performed in infertility clinics.

HBA has recently been marketed as an addition to semen analysis for predicting sperm fertilizing ability. Although its clinical value is still investigational, HBA is becoming a more popular test in many ART clinics worldwide (32). While SPA evaluates multiple steps that lead to fertilization, HBA is limited to the step before acrosome reaction (AR). Even though the SPA test evaluates acrosome reaction only indirectly, the HBA assesses HA-bound sperm which have intact acrosomes that have not undergone AR (10). After sperm capacitation any of the remaining steps that lead to fertilization can fail. The HBA test cannot predict failure of post capacitation events as the SPA can, since the purpose of SPA is to evaluate sperm fusion and decondensation as consequence of capacitation.

Previous publications have suggested that HBA may be a useful additional test to the semen analysis and the SPA test (10). The semen analysis and SPA can help give a definitive diagnosis of male infertility and HBA can be an important tool in selecting the best quality sperm used in ICSI. There is a known relationship between HA bound sperm and enhanced levels of developmental sperm maturity, including fewer chromosomal aberrations and higher sperm DNA integrity (10). Recently the FDA approved a device in which the HBA test serves as a tool in assisting an embryologist in selecting mature sperm for ICSI based on sperm binding to hyaluronan, the main constituent of the cumulus oophorous surrounding the oocyte.

Although we used small numbers of patients, data obtained from this study do not tend to support the possibility that HBA can replace SPA. Recently it has been found that the clinical predictive value of HBA for sperm fertilizing ability in vitro is limited and that HBA does not provide additional information for identifying patients with a poor fertilization rate (32). In their study Hong et al. outlined that HBA is not superior to the routine semen analysis for predicting sperm fertilizing ability (32). Freeman et al. however, concluded that SPA is a more sensitive screening tool for the prediction of IVF fertilization than the semen analysis alone (22). Furthermore in their hands the SPA test was accurate in predicting fertilization rates in IVF when performed with standardized procedures and appropriate capacitation time (22). Multiple other groups have confirmed that SPA is a reliable assay for determining the fertilizing ability of human spermatozoa (33–39).

We conclude that since HBA scores do not correlate with SPA results, the HBA test does not reduce the need for (and cannot replace) the SPA test in male partner screening prior to infertility treatment.

## Figures and Tables

**Table 1:** Semen parameters for sample population

*Parameter*	*Mean+/− SD*	*Range*
Count ( × 10^6^)	67.78 +/− 53.59	221 - 4.8
% Motility	70.54 +/− 22.73	98 - 23
Total motile	142.26 +/− 130.13	469.6 - 6.3
Kruger morphology (%)	11.85 +/− 4.45	20 - 2
SCI results	10.68 +/− 7.32	23.4 - 0
HBA score (%)	74.85 +/− 15.64	97 - 26
